# Genetic and physical mapping of anther extrusion in elite European winter wheat

**DOI:** 10.1371/journal.pone.0187744

**Published:** 2017-11-09

**Authors:** Quddoos H. Muqaddasi, Klaus Pillen, Jörg Plieske, Martin W. Ganal, Marion S. Röder

**Affiliations:** 1 Leibniz Institute of Plant Genetics and Crop Plant Research (IPK), Stadt Seeland OT Gatersleben, Germany; 2 Institute of Agricultural and Nutritional Sciences, Martin-Luther-University Halle-Wittenberg, Halle, Germany; 3 TraitGenetics GmbH, Stadt Seeland OT Gatersleben, Germany; New South Wales Department of Primary Industries, AUSTRALIA

## Abstract

The production and cultivation of hybrid wheat is a possible strategy to close the yield gap in wheat. Efficient hybrid wheat seed production largely depends on high rates of cross-pollination which can be ensured through high anther extrusion (AE) by male parental lines. Here, we report the AE capacity and elucidate its genetics in 514 elite European winter wheat varieties via genome-wide association studies (GWAS). We observed a wide range of variation among genotypes and a high heritability (0.80) for AE. The whole panel was genotyped with the 35k Affymetrix and 90k iSELECT single nucleotide polymorphism (SNP) arrays plus *Ppd-D1*, *Rht-B1 and Rht-D1* candidate markers. GWAS revealed 51 marker-trait associations (MTAs) on chromosomes 1A, 1B, 2A, 4D and 5B, with *Rht-D1* (4D) being the most significant marker. Division of whole panel according to the *Rht-D1* genotype resulted in 212 and 294 varieties harboring *Rht-D1a* and *Rht-D1b* allele, respectively. The presence of *Rht-D1a* compared to *Rht-D1b* (mutant) allele had a large phenotypic influence on AE resulting in its ~17% increase. GWAS performed on the sub-panels detected novel MTAs on chromosomes 2D, 3B and 6A with increased phenotypic variance imparted by individual markers. Our study shows that AE is a highly quantitative trait and wild type *Rht-D1a* allele greatly improves AE. Moreover, demarcating the quantitative trait loci regions based on intra-chromosomal linkage disequilibrium revealed AE’s candidate genes/genomic regions. Understanding the genetics of AE in elite European wheat and utilizing the linked markers in breeding programs can help to enhance cross-pollination for better exploitation of heterosis.

## Introduction

Harnessing the advantages of heterosis has emerged as an important strategy for improving and stabilizing yield in many crops. Heterosis, a phenomenon where a hybrid offspring exhibits superior performance compared to the parents, has been in the focus of both plant breeders and geneticists [1]. While hybrid cultivars are widely used in out-crossing species such as maize, rapeseed and rye [2], heterotic advantages in wheat have so far not been optimally realized. Much of this owes to the strict inbreeding nature of wheat. Wheat flowers having male and female organs in the same floret often does not open during flowering, forming the basis of self-pollination or cleistogamy in wheat. Industrial scale production of hybrid wheat seed demands a high level of pollen shedding outside the florets by male parents for efficient cross-fertilization of the female parents. Sufficient opening of florets at flowering time is therefore crucial to help anthers extrude, dehisce and shed pollen outside the floret.

The subject of hybrid wheat breeding has a long history [3] with a modest success [4]. A special emphasis has been given to the importance of anther extrusion (AE) for generating pools of efficient pollinators [5–8] and a more recent surge in hybrid wheat breeding also highlights the importance of high AE by male parental lines to improve cross-pollination and subsequent seed set on female parents [4, 9–13]. AE, a phenomenon in which anthers at yellow stage come out of the florets at flowering time [14], therefore, is a major contributing trait for hybrid wheat seed production. To prevent self-pollination and to achieve male sterility in female parents, the most common system for industrial scale hybrid wheat production is the use of chemical hybridization agents (CHAs) [4]. The application of CHAs renders the stamens of the female parent unfertile (male sterile). Male sterile female parents are subsequently pollinated via cross-pollination from male parental lines.

Recent positional cloning of a cleistogamous gene (c*leistogamy 1*) in barley showed that it inhibited the flower opening by reducing the size of the lodicules [15]. *Cly1* encoded a transcription factor containing two AP2 domains and a putative microRNA (miR172) targeting site. Cleavage of mRNA directed by miR172 was detectable only in a non-cleistogamous background [15]. Orthologous genes to the barley cleistogamous gene *Cly1* were detected in wheat in the subtelomeric regions of the long arms of the group 2 chromosomes [16]. These genes were designated as *TaAP2-A*, *-B* and–*D*, and had a high transcript abundance in the lodicules. Like the barley genes, the *TaAP2* mRNAs were cleaved at their miR172 sites. However, resequencing of the *TaAP2* genes showed a high degree of conservation for these genes even across different ploidy levels and no functional variants at the key miRNA172 targeting site were detected [17].

A wide range of variation for AE among wheat genotypes has been reported in recent studies [9, 10, 12, 18–21]. The AE variation can be utilized for i) efficient selection, breeding and development of high AE genotypes and ii) designing crosses between contrasting phenotypes for mapping purposes. The genetic architecture of AE or its equivalent “anther retention” has been investigated by linkage mapping [18–20, 22]. More recently, genome wide association mapping studies (GWAS) of AE on wheat genetic resources [10, 23], advanced spring breeding lines [14] and winter varieties [9] have been conducted and a strong quantitative genetic nature for this trait was revealed. GWAS is a widely used approach to establish the marker trait associations (MTAs) by harnessing the benefits of a large number of marker polymorphisms, the extent to which these markers are in linkage disequilibrium (LD) to the genes controlling the trait, long recombination history and large size of the investigated population [24–27]. The availability of single nucleotide polymorphic (SNP) markers in large numbers [28, 29] provides genome-wide variations which are exploited in GWAS. With the availability of the first draft sequence of hexaploid wheat genome [30], it also becomes possible to link the results of genetic mapping to the physical map and to identify the genomic regions underlying the detected quantitative trait loci (QTL) with the ultimate goal to determine the causal genes.

The aims of this study were i) to study variance of AE in elite European winter wheat varieties also with regard to the dwarfing gene *Rht-D1* ii) to perform GWAS of AE by using high marker density platforms and iii) to link the detected QTL to the current physical map of wheat.

## Materials and methods

### Plant material and collection of phenotypic data

An elite European winter wheat panel consisting of 514 varieties was used in this study (S1 Table). The whole panel was evaluated for its capacity of anther extrusion (AE) in field trials at the IPK-Gatersleben, Germany for two consecutive years (2015 and 2016). The individual plot size was 2 × 2 m, and each plot was split into six rows spaced 0.20 m apart. Standard agronomic wheat management practices were applied.

To estimate the AE, we first estimated the anther retention (AR; number of non-extruded anthers). AR was scored in six and ten spikes per plot in 2015 and 2016, respectively. The spikes were harvested five to seven days post-anthesis and held at -20°C. After defrosting, AR was scored by counting the anthers retained inside the lateral florets of four spikelets sampled from the central portion of the spikes (two pairs on each side). AE was calculated by subtracting the number of retained anthers from 24, since the total number of anthers housed by eight florets is 24 (4 spikelets × 2 lateral florets × 3 anthers).

### Statistical analysis of phenotypic data

We performed phenotypic analysis by fitting a linear mixed-effect model. The following model was used:

$$y_{ijk}\;=\;\mu +G_{i}+E_{j}+{\left(G\times E\right)}_{ij}+R_{\left(j\right)k}+\;e_{ijk}$$

where, *y*_*ijk*_ denotes the phenotypic record of the *i-*th genotype in the *k*-th replication of the *j-*th environment, *μ* is the common intercept term, *G*_*i*_ is the effect of the *i*-th genotype, *E*_*j*_ is the effect of the *j*-th environment, (*G* × *E*)_*ij*_ is the interaction effect between the *i*-th genotype and *j*-th environment, *R*_(*j*)*k*_ is the effect of *k*-th replication in *j*-th environment and *e*_*ijk*_ is the corresponding residual term. Note that as field trials in both years were carried out at the IPK-Gatersleben, we denote years as environments for convenience and interpretation in further analyses.

We first assumed all effects except the intercept as random to estimate the variance components. The repeatability among the replicates in individual years was calculated as $H^{2}=\frac{\sigma_{G}^{2}}{\sigma_{G}^{2}+\left(\frac{\sigma_{e}^{2}}{n_{R}}\right)}$ and the broad sense heritability between the years as $H^{2}\;=\;\frac{\sigma_{G}^{2}}{\sigma_{G}^{2}+(\frac{\sigma_{G\times E}^{2}}{n_{E}})+(\frac{\sigma_{e}^{2}}{n_{E}\times n_{R}})}$ where ${\;\sigma }_{G}^{2}$, $\sigma_{G\times E}^{2}$ and $\sigma_{e}^{2}$ denote the variance components of genotypes, genotype-by-environment interaction and the residuals, respectively. *n*_*E*_ and *n*_*R*_ denote the number of environments and number of replications, respectively.

To calculate the best linear unbiased estimates (BLUEs) of each genotype, we assumed fixed intercept and genotypic effects, whereas all other effects in the aforementioned model remained random. All the above mentioned calculations were performed in R [31] by using lme4 package [32].

### Genotyping and analysis of population structure

All 514 winter wheat varieties used in this study were genotyped with a 35k Affymetrix and a 90k iSELECT single nucleotide polymorphism (SNP) array [28, 29] which generated 35,143 and 81,587 SNP markers, respectively. We performed quality control for the SNP markers by setting the parameter of rejecting the SNPs having more than 5% missing values and heterozygotes, and the SNPs with minor allele frequency (MAF) below 0.05 on both SNP arrays. In total, quality control on SNP data resulted in 27,197 SNPs which remained in our study for the subsequent analyses. In addition to SNP genotyping, we genotyped the whole panel with three candidate gene markers (a photoperiodism gene *Ppd-D1*, and two dwarfing genes *Rht-B1* and *Rht-D1*) [33, 34].

To examine the genetic structure among the genotypes, we performed principal component analysis (PCA) based on SNP genotypes. The first two PCs were drawn two dimensionally to see the pronounced clustering among varieties. We also calculated the variance-covariance kinship among the genotypes following VanRaden (35) to see the genetic relatedness. The aforementioned estimates were performed in Genome Association and Prediction Integrated Tool (GAPIT) [36]. Furthermore, to see the hidden population sub-structuring, we used R-based STRUCTURE-like inference algorithm LEA by assuming 10 ancestral populations (*K* = 1 − 10) and using the function snmf which provides least squares estimates of ancestry proportions and estimates an entropy criterion that evaluates the quality of fit of the statistical model to the data by using a cross-validation technique. The number of ancestral populations, which best explain the genotypic data can be chosen by using the entropy criterion. We performed 10 repetitions for each *K* and the optimal repetition explaining the minimal cross-entropy value was used to visualize clustering among varieties by drawing bar plots [37, 38].

### Genome-wide association mapping

A regular linear mixed model [39] was implemented for genome-wide association studies (GWAS) as:

y = Xβ+Zu+e

where ***y*** is the vector of the BLUEs of each genotype, ***β*** is a vector containing fixed effects of the genetic marker and the intercept, ***u*** is a vector of random additive genetic effects of each genotype, ***X*** and ***Z*** are the corresponding design matrices and ***e*** is the vector of residuals. In the model, the genetic (***u***) and the residual (***e***) effects were assumed random, independent and normally distributed with null means as $u\sim N(0,K\sigma_{a}^{2})$, $e\sim N(0,{I\sigma }_{e}^{2})$. In GWAS, we corrected for the confounding effect of population structure by assigning a kinship matrix (***K***) as the variance-covariance matrix [35] for the random additive genetic effects. The association mapping scan was performed in R by using GAPIT package [36]. We set a threshold of significance of *P*<0.001 (−log_10_(*P*) ≥ 3.0) for the detection of marker-trait associations (MTAs). To check whether the model we implemented was sufficiently stringent to control the population stratification, family structure or false positives, quantile-quantile (qq) plots were drawn based on observed *versus* expected *P* values of all the SNPs at −log_10_ scale.

We studied the influence of the most significant SNPs (MS-SNPs i.e., SNPs with highest −log_10_(*P*) and largest effect) from each chromosome on the AE phenotype by taking their both alleles. Boxplots were drawn for varieties containing individual alleles (reference and variant/minor allele) of MS-SNPs.

### Linkage disequilibrium analysis and connection of significant SNPs to the genome zipper

A detailed description of the LD analysis is given in the S1 Note. Briefly, genome-wide LD analysis based on *r*^2^ measure [40] was performed between the significant SNP profiles and genome-wide SNPs. An *r*^2^ threshold of >0.20 was set to define the SNPs to be in LD (henceforth termed as LD-SNPs) with the significant SNPs. The entire chromosome-wise LD-SNPs were BLASTed [41] against the wheat genome assembly IWGSC1+popseq [30]. This resulted in the detection of the unique gene specific SNPs generating the gene identifiers (gene IDs), transcript IDs and the direction of the genes. These gene IDs were anchored onto the genome zipper [42], a resource which gives an ordered structure of the wheat genes based on synteny to well established grass genomes viz., *Brachypodium distachyon*, *Sorghum bicolor* and rice (*Oryza sativa)*.

### Visualizing the QTL transcript profiles

Anchoring the LD-SNPs onto the genome-zipper revealed putative quantitative trait loci (QTL) for AE. With the exception of chromosome 2A and 6A where not all the LD-SNPs were clustered in the same genetic region on genome zipper, all chromosome-wise LD-SNPs were generally clustered together. We extracted these genome zipper regions (QTL spanning ~3–5 cM) containing wheat genes and their syntenic genes of *Brachypodium*, *Sorghum* and rice on corresponding chromosomes. These QTL regions contained several wheat genes (genes that belong to the LD-SNPs and those in between). The transcript IDs of all these genes were taken from wheat sequence [30]. We used another publically available data base *expVIP* [43] to get the expression profiles of all these genes present in QTL in various organs of the wheat plant. The expression profiles were retrieved in transcripts per million (tpm) at log 2 scale. We divided the organs as AE related organs i.e., those which can influence AE (spikes, spikelets, stamen and pistil) and AE unrelated organs i.e., those which may not have any functional relevance to AE (roots, leaves, seedling and grain). To visualize the expression profiles, heat maps were drawn from the expression data of all the genes (transcript IDs) by using R package *ComplexHeatmap* [44].

## Results

### Intensive phenotyping results in correct estimates of variance components and heritability for anther extrusion

**Fig 1:**
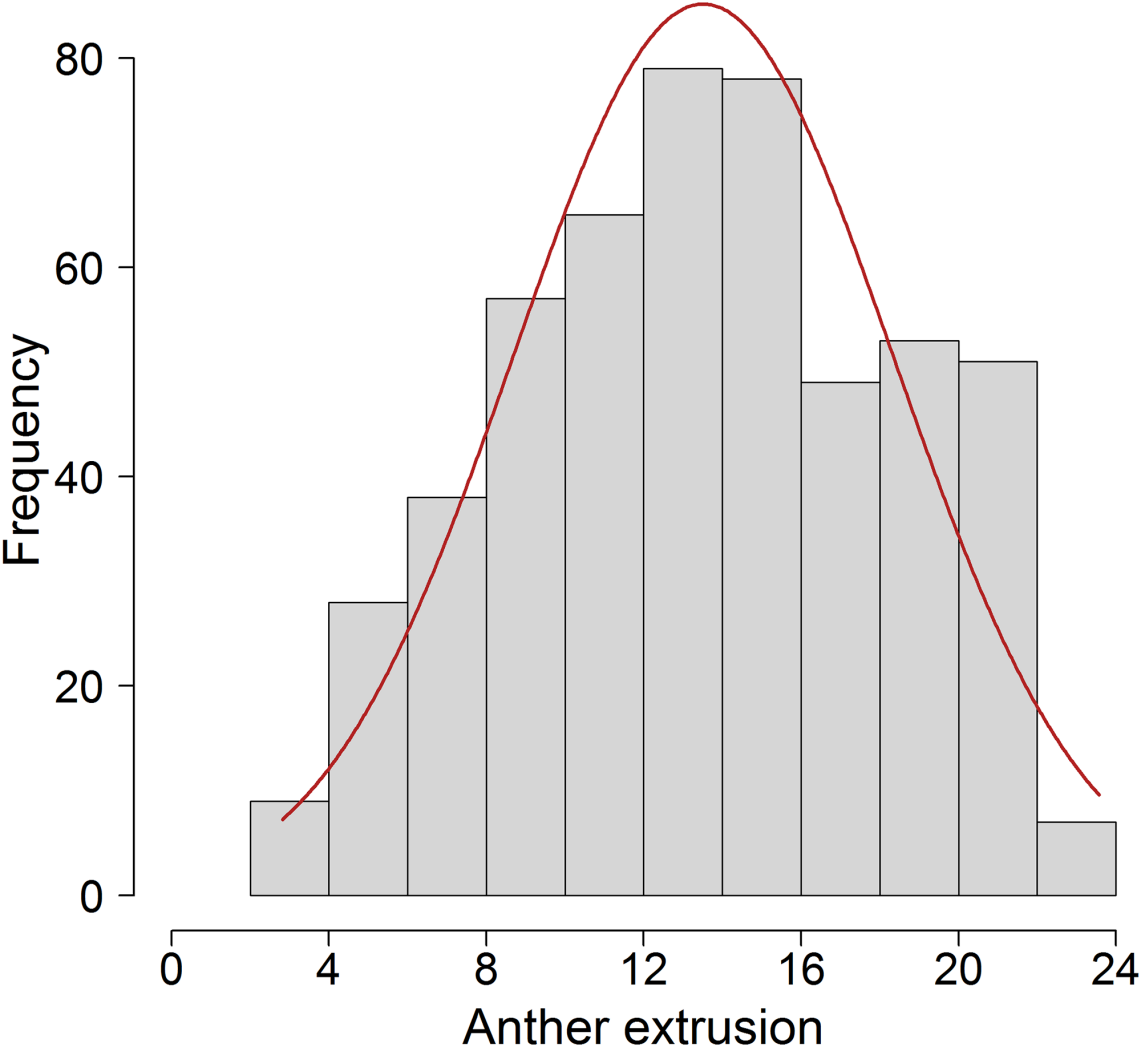
Distribution of the best linear unbiased estimates (BLUEs) of the trait anther extrusion.

Anther extrusion (AE) was scored in 514 winter wheat varieties for two years (2015 and 2016). We observed a significant positive Pearson’s product moment correlation (*r* = 0.68; *P*<0.001) between the mean AE values of both years (S1 Fig). The resulting best linear unbiased estimates (BLUEs) from both year data approximated a normal distribution ranging from 2.82 to 23.57 with a mean value of 13.51 (Fig 1). Individual variance component analysis showed that the genotypic variance ($\sigma_{G}^{2}$) principally contributed to AE (77.35%) whereas environment ($\sigma_{E}^{2}$) contributed only 13.38% with remaining variance accounted collectively by $\sigma_{G\times E}^{2}$ and $\;\sigma_{e}^{2}$. The estimates of repeatability among the replicates in individual year were high (2015 = 0.95, 2016 = 0.97). The broad sense heritability estimate between the years amounted to 0.80.

### Population structure reveals absence of genetically distinct sub-populations

**Fig 2:**
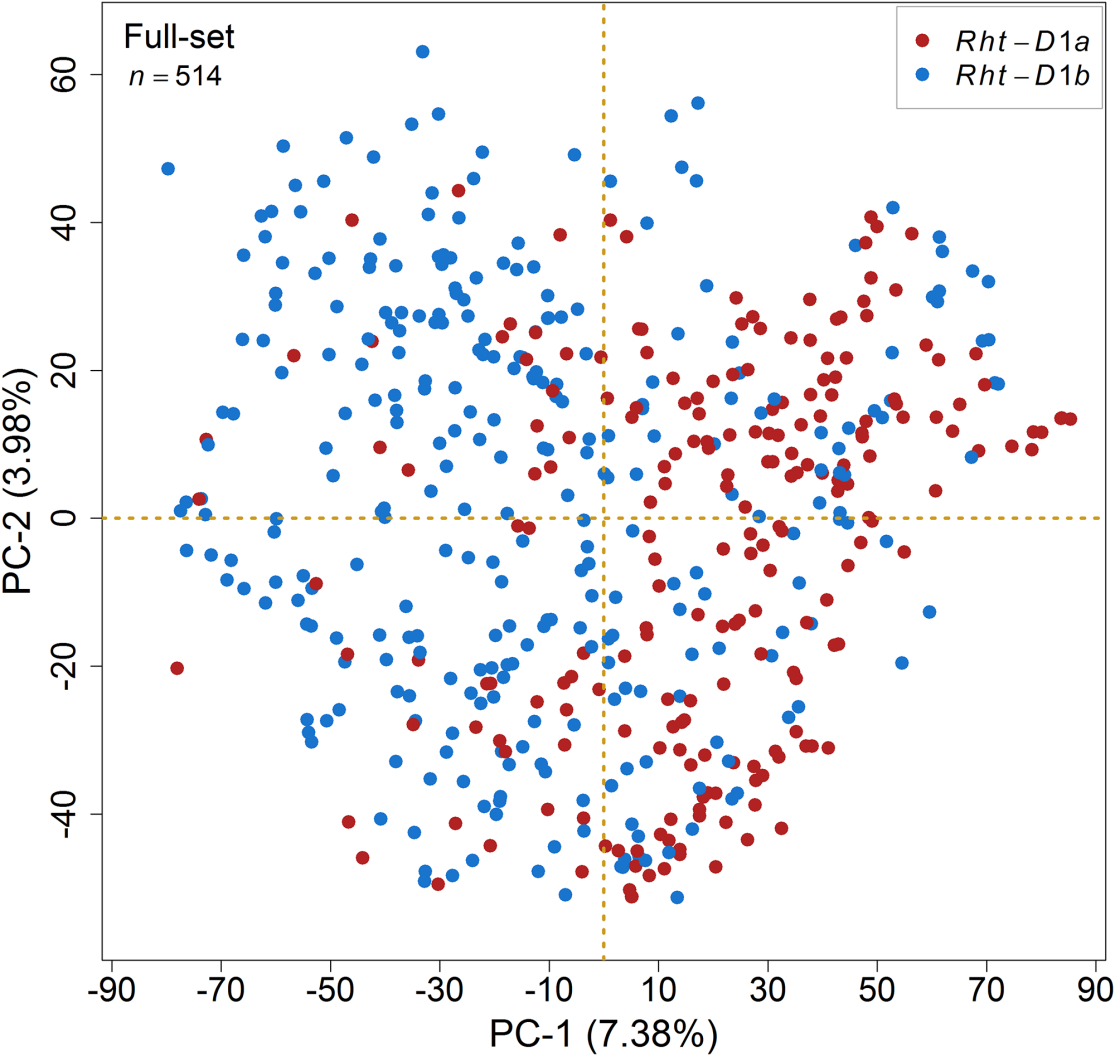
Principal component analysis (PCA) of wheat varieties based on SNP genotype explains the absence of pronounced population structure. Full-set means that the varieties harbored both *Rht-D1a* and *Rht-D1b* alleles and *n* denotes the total number of varieties used.

Extensive SNP genotyping on our panel provided 27,197 high quality SNP markers *plus* the candidate markers for three genes viz., *Ppd-D1*, *Rht-B1* and *Rht-D1*. Principal component analysis (PCA) based on SNP genotypes to examine the population structure in our panel revealed the absence of distinct sub-populations with first two PCs explaining only 11.36% of the total variation. There seems to be present, however, a substructure dividing the varieties with respect to the wildtype allele *Rht-D1a* and the mutant allele *Rht-D1b* of the dwarfing gene *Rht-D1* on chromosome 4D (Fig 2).

Additionally, we employed a STRUCTURE-like inference algorithm LEA to reveal the correct sub-populations in our panel [38] by assuming the ancestral populations (*K*) between 1 and 10, with cross-entropy and 10 repetitions. This resulted in the distinction of sub-populations but with slight entropy shift. The bar plots and the cross-entropy plot indicated three weak sub-clusters showing a change of slope in the curve at *K* = 3. The point that the curve does not reach a minimum value indicates that there might exist a slight sub-structuring in our panel. However, to accurately see the sub-population clustering, bar plots for *K* = 2–6 were drawn. Nevertheless, we could see no pronounced clustering among varieties (S2 Fig).

### GWAS reveals *Rht-D1* as a major QTL to promote AE

**Fig 3:**
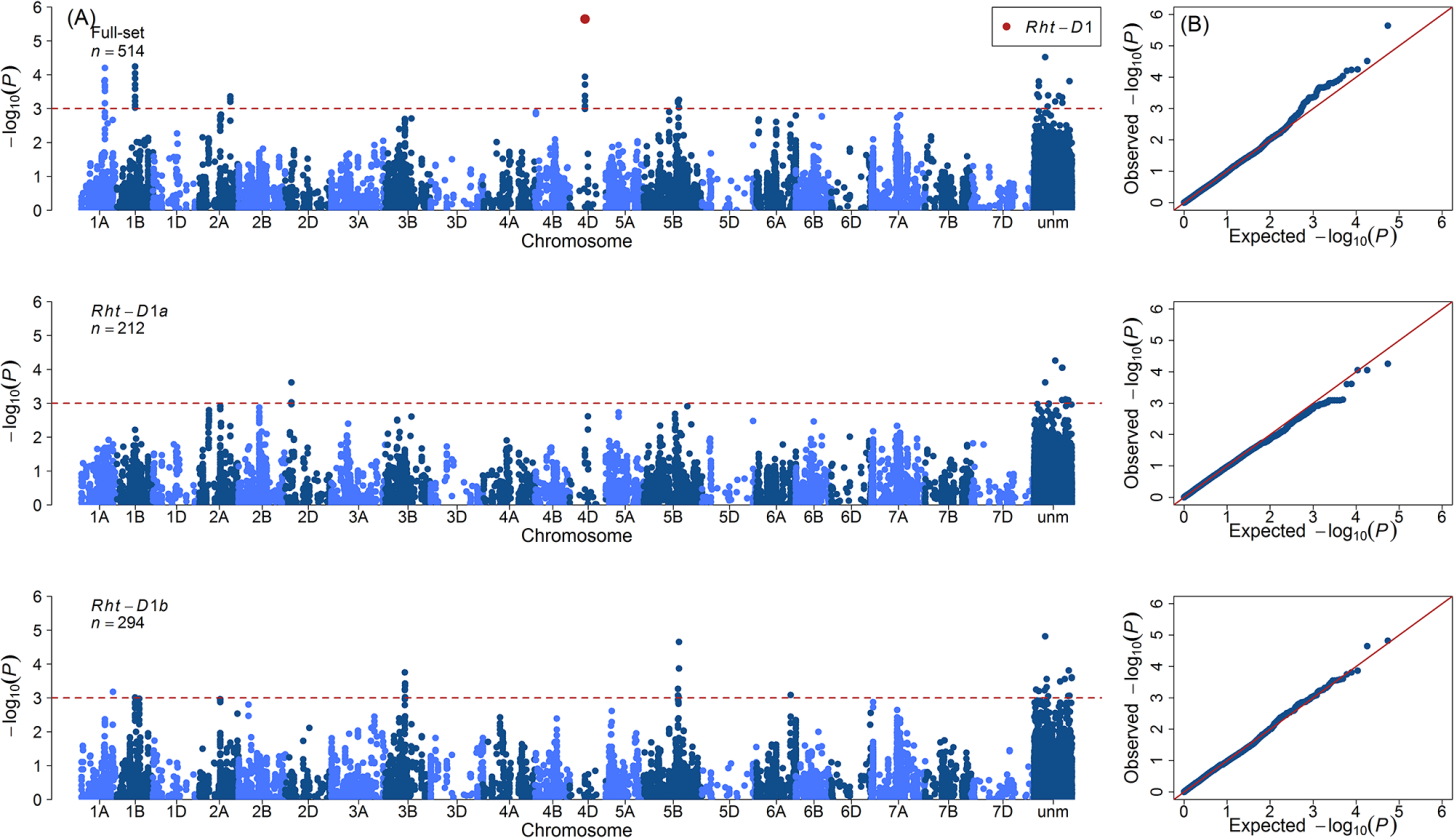
Summary of genome-wide association studies (GWAS) for anther extrusion in different wheat panels. **(A)** Manhattan plot based on linear mixed model using kinship matrix for correction of population stratification. **(B)** Quantile-quantile plot depicting expected *versus* observed *P* values at −log_10_ scale. Full-set represents the GWAS results of varieties harboring both *Rht-D1a* and *Rht-D1b* alleles. *Rht-D1a* and *Rht-D1b* set represent the GWAS results in individual panels harboring either of the alleles. *n* denotes the total number of varieties used in GWAS panels.

**Fig 4:**
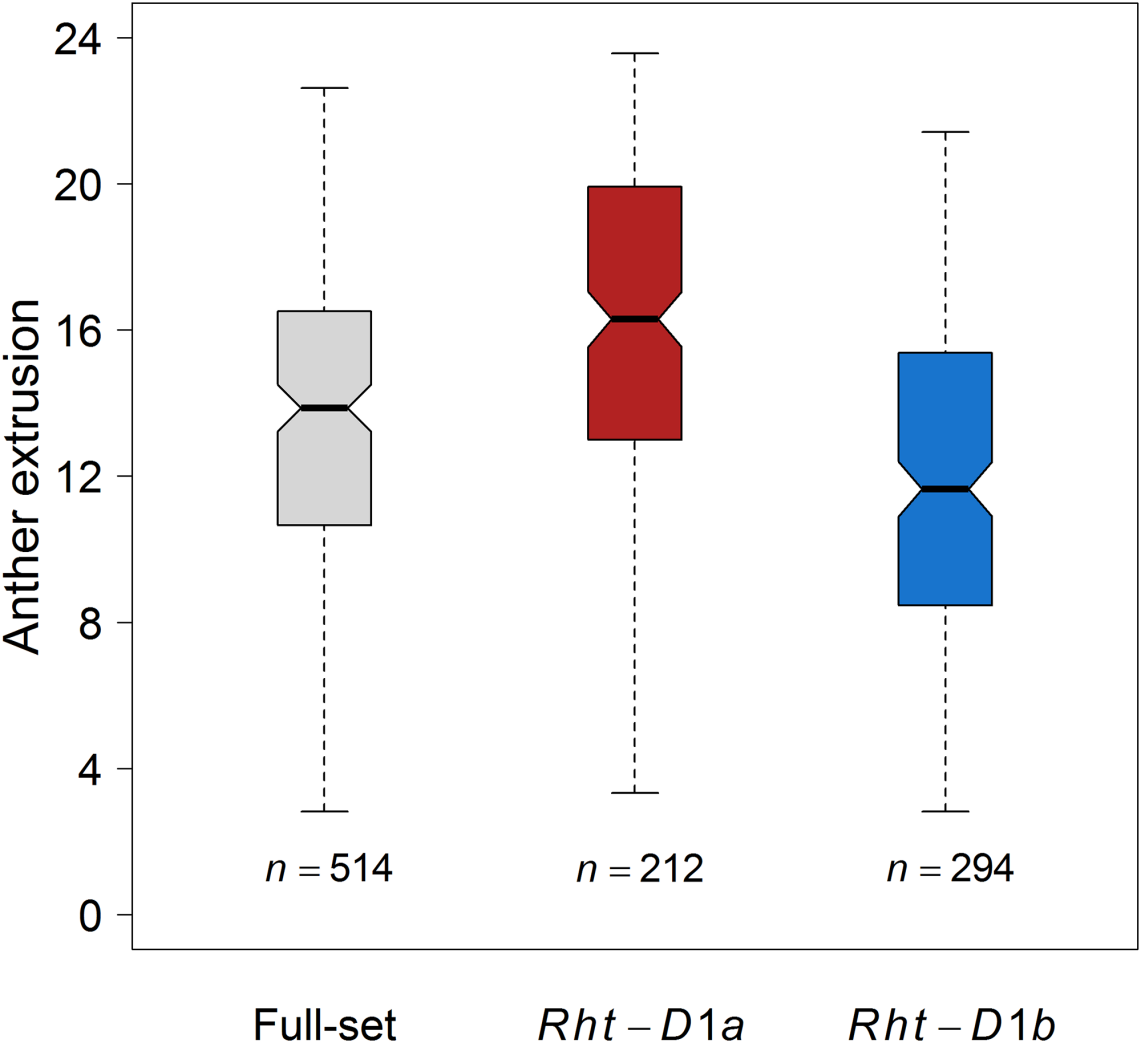
Distribution of anther extrusion in wheat panels harboring both *Rht-D1a* and *Rht*-*D1b* alleles (full-set) and panels harboring only one of the alleles. *n* denotes the number of varieties in individual panels.

**Fig 5:**
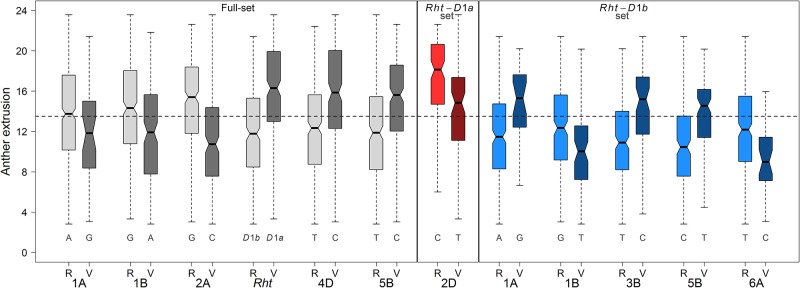
Influence of the most significant SNPs (SNPs with the largest additive effect) on AE phenotype based on GWAS conducted on panels harboring full-set of varieties and varieties harboring only *Rht-D1a* and *Rht-D1b* alleles. Horizontal dotted line marks the mean of AE BLUEs. R and V denote the varieties harboring reference (major) and variant (minor) alleles, respectively.

In total, 51 marker-trait associations (MTAs) underlying the genetic architecture of AE were identified. The significant MTAs were detected on chromosomes 1A, 1B, 2A, 4D and 5B with *Rht-D1* on chromosome 4D being the most significant (−log_10_(*P*) = 5.64) marker (Fig 3). Of the significant MTAs, 39 and 12 had negative and positive additive effects on the trait AE, respectively. The strong association of *Rht-D1* with AE led us to divide the full set of varieties into two sub-panels with the varieties harboring either the wild type *Rht*-*D1a* (212 varieties) or the mutant allele *Rht*-*D1b* (294 varieties) and to perform a second round of GWAS on the individual panels. The phenotypic distribution of AE in these sub-panels showed that the *Rht-D1a* panel had a mean value 15.91 compared to the mean of 11.81 for the *Rht-D1b* panel (Fig 4). Moreover, to see if the individual panels had a pronounced population structuring, we performed PCA, which showed the absence of distinct sub-populations (S3 Fig). A second round of GWAS performed individually on *Rht-D1a* and *Rht-D1b* panels resulted in the detection of 14 and 31 MTAs, respectively. The GWAS model parameters were remained unchanged. The MTAs identified from the *Rht-D1a* panel were present on chromosome 2D, while those from the *Rht-D1b* panel were present on chromosomes 1A, 1B, 3B, 5B and 6A. The MTAs detected in these panels on chromosomes 2D, 3B and 6A were novel and remained undetected when GWAS was performed on the full-set (Fig 3 and S2 Table). While the *R*^2^ values for the full set ranged from 1.31% to 2.47%, the sub-panels had increased *R*^2^ values ranging from 2.54% to 4.54% (Table 1 and S2 Table). Boxplots of the most significant SNPs according to both alleles (reference and variant/minor allele) revealed the influence of the minor (variant) alleles on AE phenotype (Fig 5). Beside *Rht-D1* and linked markers on chromosome 4D, markers on chromosome 5B showed a positive effect of the minor allele in the full-set of varieties, while for the *Rht-D1b* sub-set markers on chromosomes 1A, 3A and 5B had positive effects on AE.

**Table 1:** List of most significant SNPs (MS-SNPs) representing QTLs on individual chromosomes.

MS-SNP	Chr.	Pos.	GWAS set	nobs	−log_10_(*P*)	maf	effect	%*R*^2^
AX_94914663	1A	72.10	full-set	514	4.20	0.08	-1.454	1.75
RAC875_c12348_720	1A	97.10	*Rht-D1b*	294	3.18	0.09	1.330	2.64
AX_94426392	1B	56.40	full-set	514	4.25	0.30	-1.006	1.78
AX_94396447	2A	95.80	full-set	514	3.36	0.38	-0.742	1.35
Kukri_c102346_668	2D	18.60	*Rht-D1a*	212	3.61	0.44	-1.086	4.54
AX_94995589	3B	59.75	*Rht-D1b*	294	3.75	0.22	1.305	3.20
*Rht-D1*	4D	-	full-set	514	5.64	0.42	1.017	2.47
RAC875_rep_c105718_585	4D	47.00	full-set	514	3.94	0.36	0.849	1.63
BobWhite_c28333_454	5B	108.70	full-set	514	3.26	0.47	0.783	1.31
Ra_c3533_880	6A	106.30	*Rht-D1b*	294	3.08	0.11	-1.220	2.54

MS-SNP = most significant SNP from each QTL having largest additive effect and highest −log_10_(*P*) value

effect = effect pertaining to the minor allele

nobs = number of varieties used in each GWAS set

maf = minor allele frequency

%*R*^2^ = % phenotypic variance imparted by corresponding SNP

### Genome-wide LD analysis and connection to genome zipper help define the QTL regions for AE

Genome-wide LD analysis based on *r*^2^ measure (Weir, 1996) between significant SNP profiles and genome-wide SNPs resulted in the detection of 330 LD-SNPs. The sequences of all the LD-SNPs blasted (BLAST) against wheat genome assembly (IWGSC1+popseq) resulted in 120 unique gene identifiers (gene IDs) on all chromosomes along with their transcript IDs and direction information (S2 Table). These gene IDs were anchored onto the genome zipper, a resource which provides an ordered scaffold of wheat genes based on synteny to the well-established genomes of *Brachypodium distachyon*, *Sorghum bicolor* and rice (*Oryza sativa*). With this scheme, we could anchor most of the genes (or LD-SNPs) onto the genome zipper on respective chromosomes. As assumed, the SNPs with high *r*^2^ values were co-segregating as they clustered in the same genetic region on respective chromosomes of genome zipper (S3 Table).

### Study of the transcript profiles can help spot candidate genes for AE

The study of the transcript-profiles of all the genes in the QTL genetic regions of genome zipper revealed that some genes were expressing exclusively or relatively higher in the AE related organs (S4 Fig). The study of expression patterns of the genes from a particular region may establish an approach to spot candidate genes for other traits as well. For example, the transcript profile of the gene *Traes_2AL_5BA7E2623*.*1* revealed its expression at relatively higher amount in flowering as compared to non-flowering organs. This gene being present on chromosome 2A was in LD with the gene corresponded by the MS-SNP on chromosome 2A. The functional annotation revealed that this is an AP2-like ethylene responsive transcription factor (S2 Table). Interestingly, these AP2-like transcription factors are described to have functions in lodicule development and floret opening in barley (*Cly1*) and wheat [15–17].

## Discussion

### High cross-pollination rates are required for hybrid wheat seed production

Stable and heritable performance of beneficial traits is required for their appropriate biological understanding and utilization in efficient breeding of crops. However, development and deployment of homogeneous elite wheat varieties has required strict inbreeding in wheat which is a bottleneck in finding suitable pollinators. Breeding for hybrid wheat demands effective pollinators (out-crossers or male parental varieties) with stable performance across years. Therefore, defining the pools of male parental varieties with high cross-pollination capacities will serve in efficient hybrid wheat breeding.

### Phenotypic selection of AE is possible with high accuracy

Anther extrusion (AE) is a major contributing trait for effective cross-pollination to improve hybrid wheat seed production. Our findings suggest that AE is a phenotypically diverse trait and varies widely among the varieties. The phenotypic distribution of AE suggests its strong quantitative nature. High genetic variance coupled with high repeatability and broad sense heritability estimates suggest that the method for AE phenotyping is reliable and the selection of AE is possible with high accuracy. These estimates are in accordance with recently published reports based on different wheat panels, methods of AE estimations and environments [9, 10, 12, 14, 18, 19]. Moreover, the strong heritable nature of AE proposes that its further genetic analyses for effective detection of quantitative trait loci (QTL) will not be compromised which is often hampered by low-quality phenotypic data [45].

### High marker density coupled with rapid LD decay help examine the complex interplay of genetic variants

High resolution mapping demands a reasonable size of mapping population, a sharp decay in LD, high density of molecular markers (genetic variation) and the extent to which these markers are in linkage disequilibrium (LD) to the causative genes [24, 46]. This approach entails the principal that markers are in close vicinity to the gene controlling the phenotype and the likelihood of co-inheritance of marker *plus* causative gene is greater than expected under independent assortment [25]. Therefore, it warrants that the likelihood of finding the causative allele is directly proportional to the size of mapping population, genome-wide marker density, marker polymorphism and rapid LD decay. In our study, the applied population size, marker density of >27,000 SNPs and a sharp decline in LD suggested that the power of GWAS was not compromised.

### GWAS suggests that AE is controlled by joint action of several genes

Although it was previously suggested that AE has a low heritability and is mainly controlled by a few, possibly, two genes [7] our results contradict these findings. The possible reasons are that the authors determined the AE in green house, used small population and worked on spring wheat. However, recent literature suggest that AE is controlled by the concerted action of several small to modest effect loci [9, 10, 18–20] which is in line with our findings. In our GWAS, we detected several MTAs which consisted of nine QTL, in total. The QTL on chromosome 4D harbored the most significant MTA with the candidate gene marker *Rht-D1a*. *Rht-D1a* is the wild type allele for the wheat reduced height (*Rht*) gene [47]. The presence of *Rht-D1a* allele compared to *Rht-D1b* (mutant) had a large phenotypic influence on AE resulting in ~17% increase, as the varieties harboring *Rht-D1a* and *Rht-D1b* alleles showed an average AE of 15.91 and 11.81, respectively. Similar results were obtained by Boeven, Longin (9). The remaining QTLs generally showed small effects, influencing AE either positively or negatively and imparted a minor phenotypic variance. The comparison of QTL intervals with previously published studies is difficult due to several problems e.g., the use of dissimilar mapping populations and molecular markers. Moreover, the comparison among previously published QTL shows that most of the QTL do not coincide both in linkage and association mapping studies. Based on these results, it seems that AE greatly depends on the mapping population background. A recent study on AE using winter wheat and SNP markers suggested several AE QTL [9]. In our study chromosome 1B harbored a QTL (~48–58 cM) signified by SNP “*AX_94426392*”. Boeven, Longin (9) reported a QTL on chromosome 1B represented by SNP “*wsnp_Ex_c5101_9053178*”. Our intra-chromosomal LD analysis shows that both of these SNPs were in LD (*r*^2^ = 0.53) with each other, suggesting that they might comprise the same QTL. Similarly *Rht-D1* was the most significant marker. The comparison of all other QTL regions suggested no connection.

### *Rht-D1a* is a likely candidate QTL for improved AE

Recently, it was reported that plant height in wheat which is mainly controlled by *Rht* genes (*Rht-B1* and *Rht-D1*) influences AE [18–20, 22]. Our GWAS also revealed *Rht-D1a* as the most significant marker associated with AE. The wild-type *Rht-1* genes (*Rht-B1a* and *Rht-D1a*) encode DELLA proteins which are sensitive to gibberellin hormones (GAs). Conversely, the mutant alleles (*Rht-B1b* and *Rht-D1b*) encode forms of DELLA proteins which are relatively insensitive to GAs [47, 48]. Bioactive forms of GAs promote plant growth and their repression by *Rht-1b* alleles results in reduced height. Coupled with improved inputs of fertilizers, introduction of *Rht-D1b* allele in wheat has uplifted yield since the onset of the Green Revolution by reducing plant height by about 17% and increasing the assimilate partitioning and consequently harvest index [49]. In addition to plant height, GAs in both crop and model species regulate several floral development processes e.g., flower induction, pollen development, pollen tube growth, stamen development, filament extension, anther development and exertion [50–52]. Recently, it was reported that GAs are most likely involved in establishing anther extrusion patterning in barley spikes [53]. Based on the association of *Rht-D1a* and its pronounced positive effect on AE phenotype, it seems that *Rht-D1a* is a likely candidate gene with a prominent pleiotropic effect on AE. Increased sensitivity to GAs seems to be the reason of high AE in varieties harboring *Rht-D1a*. However, possibility of other genes or QTL effecting AE in wheat cannot be ruled out because GWAS identified several minor effect loci as well. For example, prevalence of *Rht-D1b* compared to *Rht-B1b* is quite high in elite European winter wheat varieties [54] which is why no association of AE with *Rht-B1* was found. These results are in agreement with recent bi-parental and association studies where *Rht-D1a* alleles were highly associated with increased AE in wheat [9, 19, 22]. It also seems that the introduction of *Rht*-*D1b* and its homoelocus *Rht*-*B1b* to develop the short statured varieties led to improved inbreeding/self-pollination as these alleles confer dwarfism by producing more active forms of GA repressors compared to their counterparts.

AE is a trait to improve male parents in hybrid wheat breeding and selection of varieties based on *Rht-D1a* will help to i) produce relatively taller male plants and ii) improve pollen shedding by male parents. Taller male plants are required for ease in pollen shed on male part and active reception of pollen on stigma, on female part. Furthermore, pollen shed by taller male parents can travel longer distances.

### Demarcating the critical QTL regions based on SNP-LD and the study of genes/ transcript content is an appropriate method for spotting the putative candidate genes in wheat

GWAS performed on a diverse and extensive set of genotypes takes the advantage of long recombination history and smaller LD decay [24]. Bi-parental populations segregating for particular traits, on the other hand, have narrow genetic base and far shorter recombination history. Moreover, time and difficulty to develop these populations remain chief obstacles for breeders. Due to fewer recombinations, bi-parental populations used in linkage mapping studies have lower-resolution *versus* association mapping studies. This indicates that the association studies are particularly useful for dissection of complex traits to capture maker alleles located close to causal variant from a broader genetic base [25, 27, 55].

QTL regions can be demarcated by estimating the intra-chromosomal LD among the SNPs i.e., estimating the LD (*r*^2^) between the marker with largest effect and the remaining markers on the same chromosome. Significant markers with large effects on the phenotype mostly act as proxies (by virtue of being in LD) for the causal genetic variant [55]. Demarcating the QTL regions by anchoring the markers (most significant and those in LD with it) on chromosome (gene order given in genome-zipper [42]) can help to study the genes located in those QTL regions. Moreover, annotated functions, transcript profiles and expression level of the genes in demarcated QTL regions supply information about their possible role. In our study, we noticed a sharp decline in LD with an average LD of *r*^2^ = 0.20 between adjacent SNPs with a 10% quantile dropping down to 0.001. This indicates that on one hand the marker density across the wheat genome can be increased and on the other hand, the markers with LD >0.20 can be considered as one linkage block or critical QTL region. By adopting this approach, we could demarcate the specific regions on the chromosomes harboring significant MTAs by aligning all significant and LD-SNPs (those in LD to the MS-SNP) on individual chromosomes. The annotated functions and expression profiles of some of the genes in critical QTL regions suggested their involvement in GA production, stamen, anther and floral expansion. For example, genes *Traes_1AL_8D078BE99*.*1*, *Traes_2AL_AC14A6352*.*1*, *Traes_4DS_4C358A987*.*1*, *Traes_4DS_7394D3FBE*.*1* and *Traes_5BL_0333145DD*.*1* expressed either exclusively or at relatively fairly high amount in stamens as compared other organs. The functional annotations of these genes suggest that they mainly belong to protein kinase and synthase families which are known to have roles in various flowering related processes [56]. Moreover, the differential expressions of AP2-like ethylene-responsive transcription factors in an AE QTL on chromosome 2A propose that they are involved in flowering related processes.

## Conclusions

Hybrid wheat breeding is attractive to both breeders and plant geneticists due to its potential as an alternative to break the yield barriers and understanding the genetic underpinnings of heterosis. Hybrid wheat seed set depends upon the efficient cross-pollination which is hampered by wheat’s strict inbreeding nature. Anther extrusion (AE) in wheat provides the basis for efficient out-crossing. Our results show that AE in elite European winter wheat has wide variation and is genetically stable. Therefore, strong selection response for AE can be expected in breeding programs. Our GWAS results demonstrate the quantitative nature of AE and that *Rht*-*D1a* is a major QTL to improve AE. Further understanding of pleiotropic effect of *Rht*-*D1* on AE in wheat can help to improve AE for efficient cross-pollination.

## Supporting information

S1 Note Linkage disequilibrium analysis to define the genetic regions in wheat. (PDF)

S1 Table List of varieties and phenotypic information. (XLSX)

S2 Table List of LD-SNPs. (XLSX)

S3 Table Anchoring the LD-SNPs onto the genome zipper. (XLSX)

S1 Fig Box and whisker plots show the distribution of the mean values of the trait anther extrusion (AE) in 2015 and 2016 and their best linear unbiased estimates (BLUEs). (PDF)

S2 Fig Population structure analysis of whole wheat panel (full-set) based on SNP genotypes. Bar plots show the existence of three to five weak sub-populations. The minimal cross-entropy plot shows slight sub-structuring within the panel.
(PDF)

S3 Fig Principal component analysis (PCA) on sets of varieties harboring either *Rht-D1a* or *Rht-D1b* alleles. *n* denotes the number of varieties present in individual set.
(PDF)

S4 Fig Expression profiles of the wheat genes in transcript-per-million (tpm) at log 2 scale. Color key is given in the figure with white marking no expression of genes and purple as highest expression. All genes (transcript IDs) are taken from the QTL genetic regions from genome zipper from corresponding chromosomes. Category refers to the organ categories based on AE-relatedness (blue) and un-relatedness (green).
(PDF)
