# Supplementary material for: Genetic and physical mapping of anther extrusion in elite European winter wheat
Source: PLoS One. 2017 Nov 9;12(11):e0187744. doi: 10.1371/journal.pone.0187744 (PMC5679578; doi:10.1371/journal.pone.0187744)
Supplement: S1 Note — (PDF) [file pone.0187744.s001.pdf]

Genetic and physical mapping of anther extrusion in elite European winter wheat

Quddoos H. Muqaddasi [1*], Klaus Pillen [2], Jörg Plieske [3], Martin. W. Ganal [3] and Marion S. Röder [1]

S1 Note. Linkage disequilibrium analysis to define the genetic regions in wheat

Single nucleotide polymorphism (SNP) genotyping data contained $27{,}197$ SNP loci, out of which $10{,}949$ (40.26%) were mapped according to mapping population used by international triticeae mapping initiative (ITMI) whereas $16{,}248$ (59.74%) SNPs were unmapped. GWAS identified 96 significant MTAs $(-\log_{10}(P) \geq 3.0)$ in all three GWAS sets (51 MTAs in full-set containing all the genotypes; and 14 and 31 MTAs in two sets of genotypes containing *Rht-D1a* and *Rht-D1b* allele, respectively). Of these 96 MTAs, 43 (44.79%) were unmapped SNPs.

To allocate the unmapped SNPs to chromosomes, we followed a two-step approach based on the linkage disequilibrium (LD) among the SNPs. To calculate LD, we adopted an r^2 measure as described in Weir (1996).

Step one: Linkage disequilibrium analysis among significant SNPs

We calculated LD (r^2) among all the 96 significant SNPs (53 mapped and 43 unmapped). This allowed us to find unmapped significant SNPs in LD $(r^2 > 0.20)$ to the mapped SNPs on chromosomes. This strategy resulted in allocation of 21 unmapped significant SNPs to the chromosomes. As a positive control to check whether the unmapped markers were mapped on the correct chromosomes, we checked for additional published resources for both 35k (Allen et al, 2016) and 90k (Wang et al, 2014) SNP arrays. Mapping populations different to ours have been used in these resources to map the SNPs to the chromosomes. We found a high congruence $(> 99\%)$ between mapping according to these resources and ours in terms of chromosomes to which the SNPs are mapped. However, since the mapping populations used in both resources are dissimilar; though the chromosomes are same, the centimorgan (cM) positions assigned to the SNPs significantly differ from each other. Nevertheless, the use of these mapping resources as a positive control to

check whether the unmapped SNPs were allocated to the correct chromosomes based on their LD values revealed the correct mapping of the unmapped SNPs.

Step two: Genome-wide LD analysis

To check if other genome-wide SNPs were in LD to the significant SNPs ($-\log_{10}(P) \geq 3.0$), we performed a genome-wide LD analysis. Of the mapped significant SNPs, we took the most significant SNPs (MS-SNPs; SNPs with highest $-\log_{10}(P)$ from each chromosome) and calculated the LD (r^2) values in the whole genome (against $27{,}197$ SNPs). In total, 330 SNPs were in LD ($r^2 > 0.20$) to the MS-SNPs which we termed as LD-SNPs. Again, the use of positive-control (other published mapping resources) revealed that $\sim 99\%$ of the SNPs shared the same chromosome based on different mapping resources.
