# Supplementary material for: Genetic and physical mapping of anther extrusion in elite European winter wheat
Source: PLoS One. 2017 Nov 9;12(11):e0187744. doi: 10.1371/journal.pone.0187744 (PMC5679578; doi:10.1371/journal.pone.0187744)
Supplement: S1 Fig — (PDF) [file pone.0187744.s005.pdf]

Genetic and physical mapping of anther extrusion in elite European winter wheat

Quddoos H. Muqaddasi [1*], Klaus Pillen [2], Jörg Plieske [3], Martin. W. Ganal [3] and Marion S. Röder [1]

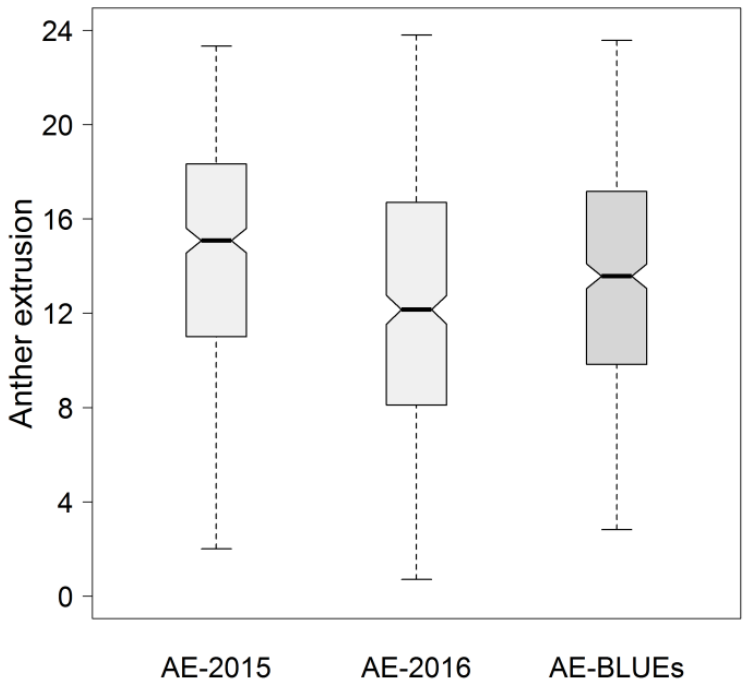

S1 Fig. Box and whisker plots show the distribution of the mean values of the trait anther extrusion (AE) in 2015 and 2016 and their best linear unbiased estimates (BLUEs).
