# Supplementary material for: Genetic and physical mapping of anther extrusion in elite European winter wheat
Source: PLoS One. 2017 Nov 9;12(11):e0187744. doi: 10.1371/journal.pone.0187744 (PMC5679578; doi:10.1371/journal.pone.0187744)
Supplement: S2 Fig — Bar plots show the existence of three to five weak sub-populations. The minimal cross-entropy plot shows slight sub-structuring within the panel. (PDF) [file pone.0187744.s006.pdf]

Genetic and physical mapping of anther extrusion in elite European winter wheat

Quddoos H. Muqaddasi [1*], Klaus Pillen [2], Jörg Plieske [3], Martin. W. Ganal [3] and Marion S. Röder [1]

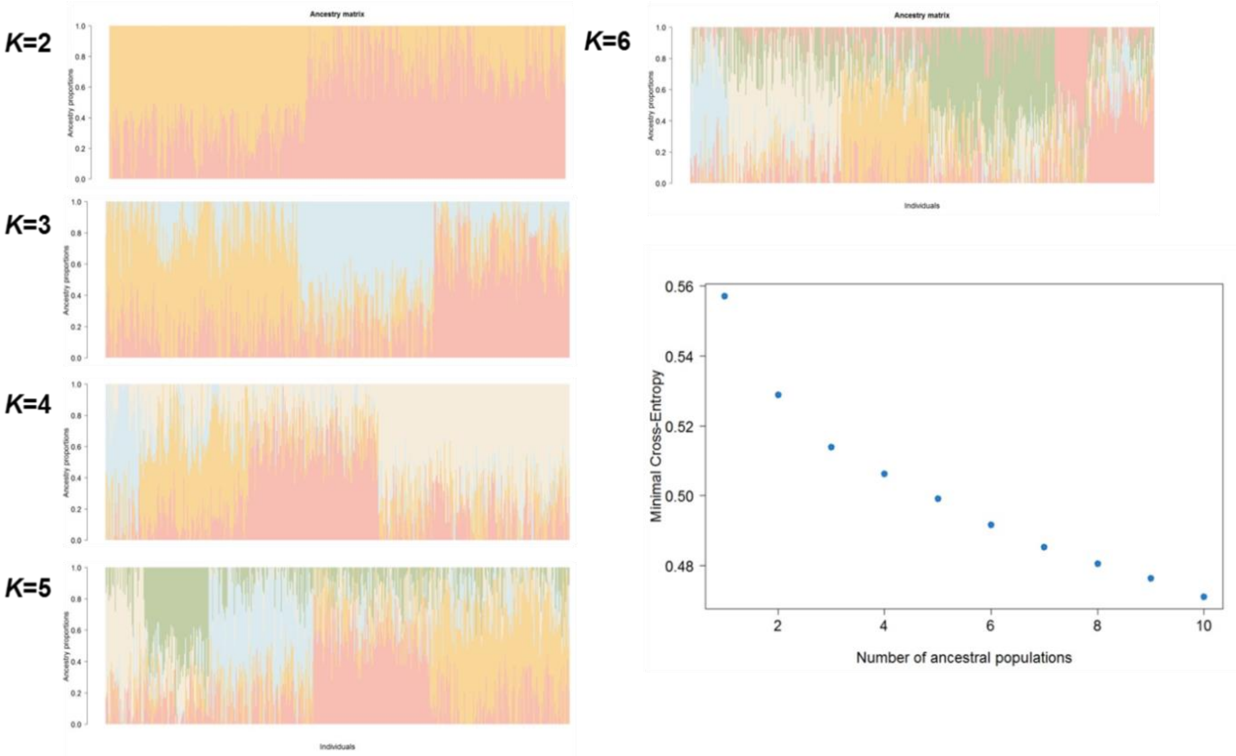

S2 Fig. Population structure analysis of whole wheat panel (full-set) based on SNP genotypes. Bar plots show the existence of three to five weak sub-populations. The minimal cross-entropy plot shows slight sub-structuring within the panel.
