# Supplementary material for: Genetic and physical mapping of anther extrusion in elite European winter wheat
Source: PLoS One. 2017 Nov 9;12(11):e0187744. doi: 10.1371/journal.pone.0187744 (PMC5679578; doi:10.1371/journal.pone.0187744)
Supplement: S3 Fig — n denotes the number of varieties present in individual set. (PDF) [file pone.0187744.s007.pdf]

Genetic and physical mapping of anther extrusion in elite European winter wheat

Quddoos H. Muqaddasi [1*], Klaus Pillen [2], Jörg Plieske [3], Martin. W. Ganal [3] and Marion S. Röder [1]

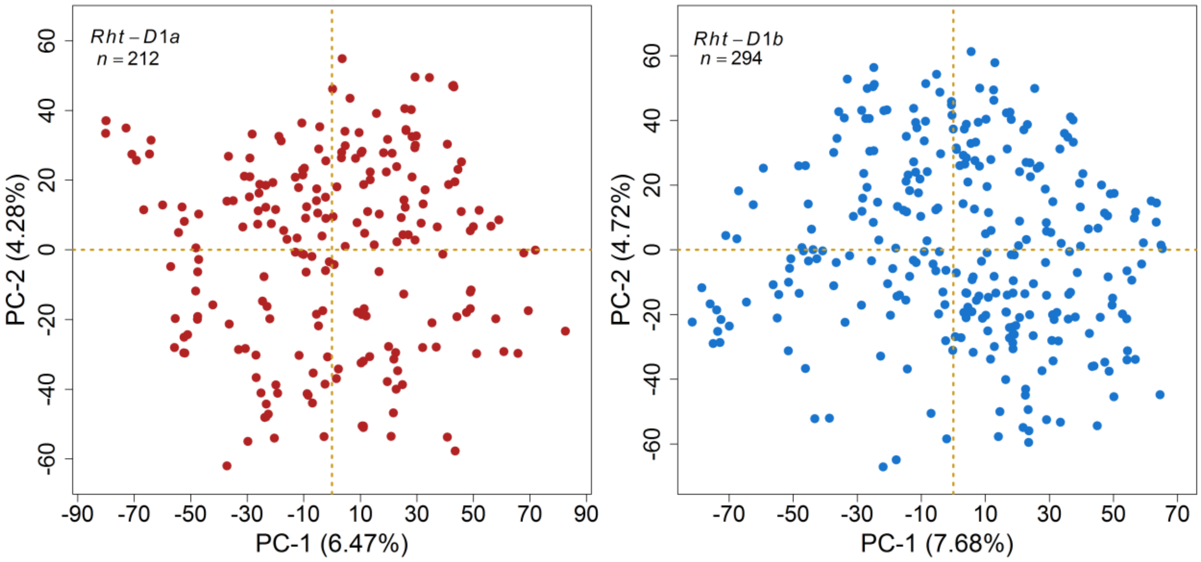

S3 Fig. Principal component analysis (PCA) on sets of varieties harboring either *Rht-D1a* or *Rht-D1b* alleles. *n* denotes the number of varieties present in individual set.
