# Supplementary material for: Genetic and physical mapping of anther extrusion in elite European winter wheat
Source: PLoS One. 2017 Nov 9;12(11):e0187744. doi: 10.1371/journal.pone.0187744 (PMC5679578; doi:10.1371/journal.pone.0187744)
Supplement: S4 Fig — Color key is given in the figure with white marking no expression of genes and purple as highest expression. All genes (transcript IDs) are taken from the QTL genetic regions from genome zipper from corresponding chromosomes. Category refers to the organ categories based on AE-relatedness (blue) and un-relatedness (green). (PDF) [file pone.0187744.s008.pdf]

Genetic and physical mapping of anther extrusion in elite European winter wheat

Quddoos H. Muqaddasi [1*], Klaus Pillen [2], Jörg Plieske [3], Martin. W. Ganal [3] and Marion S. Röder [1]

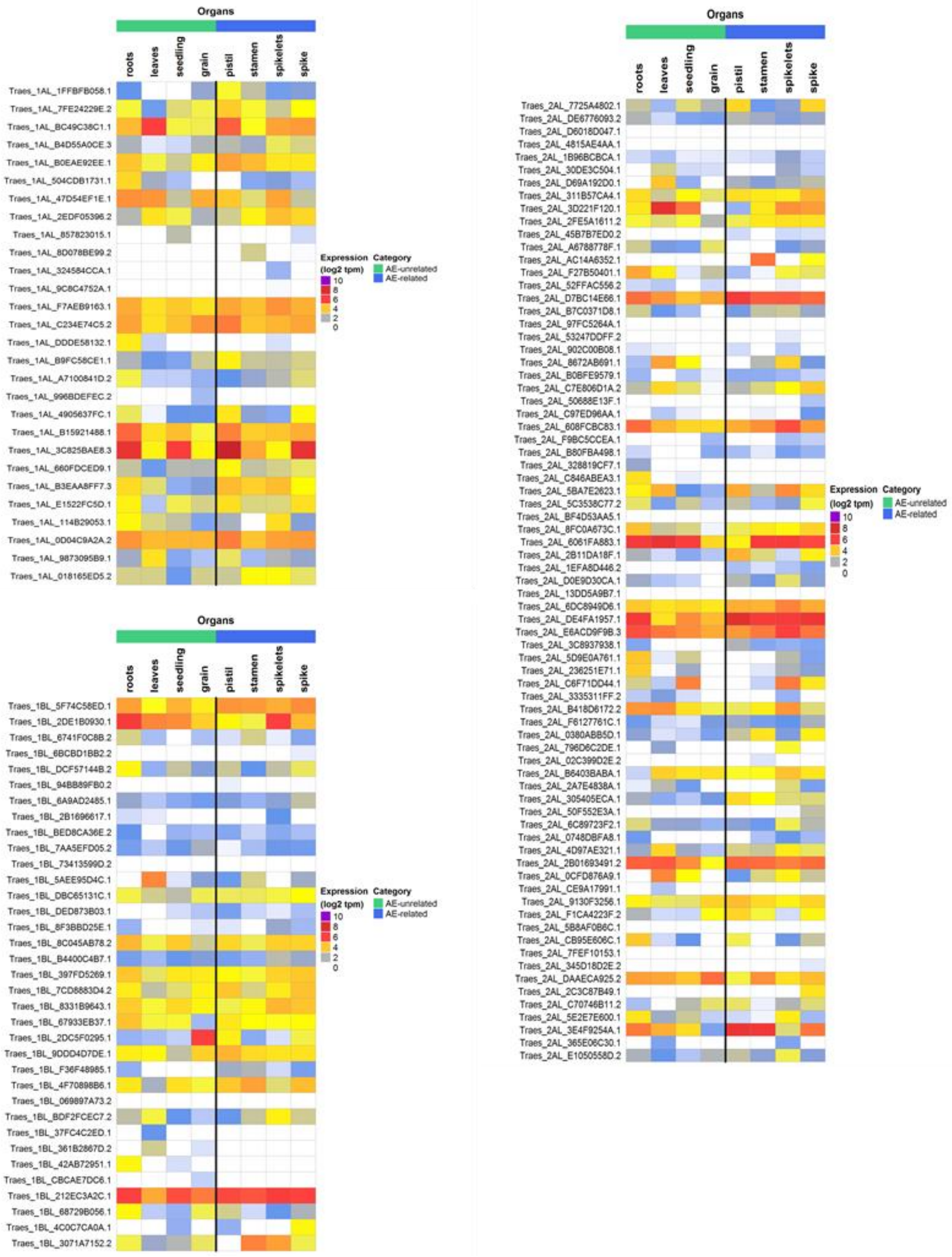

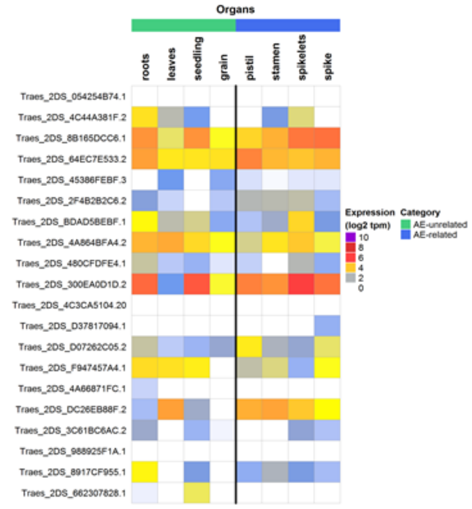
Organs
roots
leaves
seedling
grain
pistil
stamen
spikelets
spike
Expression (log2 tpm)
10
8
6
4
2
0
Category
AE-unrelated
AE-related

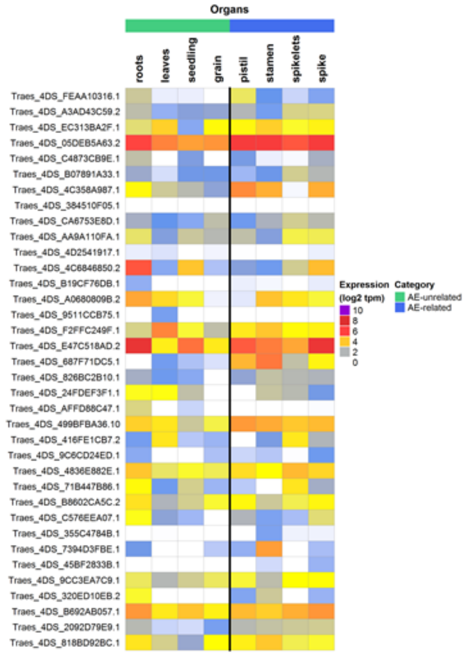
Organs
roots
leaves
seedling
grain
pistil
stamen
spikelets
spike
Expression (log2 tpm)
10
8
6
4
2
0
Category
AE-unrelated
AE-related

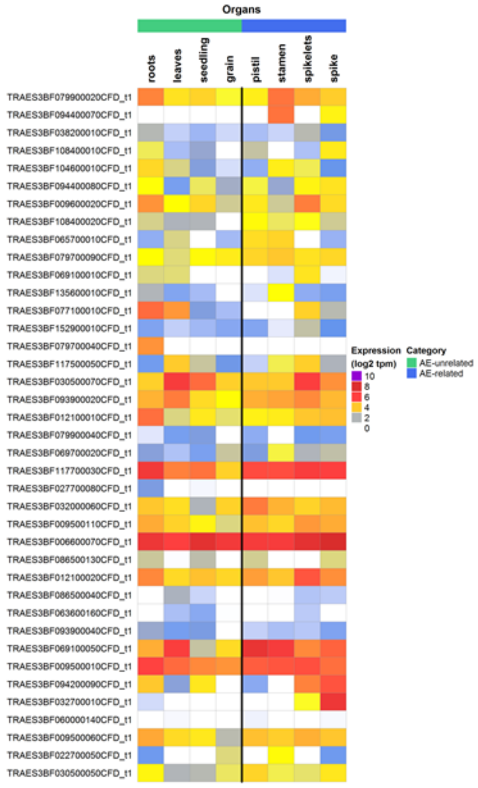
Organs
roots
leaves
seedling
grain
pistil
stamen
spikelets
spike
Expression (log2 tpm)
10
8
6
4
2
0
Category
AE-unrelated
AE-related

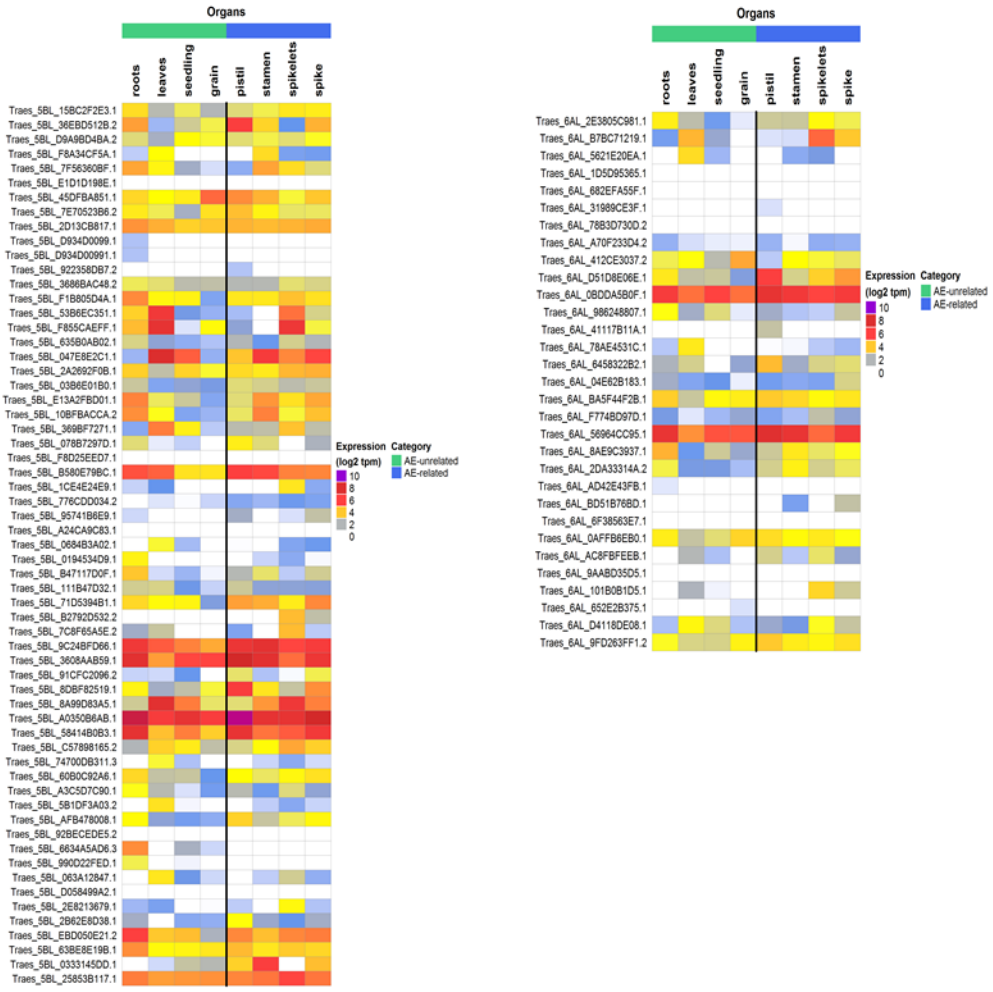

S4 Fig. Expression profiles of the wheat genes in transcript-per-million (tpm) at $\log 2$ scale. Color key is given in the figure with white marking no expression of genes and purple as highest expression. All genes (transcript IDs) are taken from the QTL genetic regions from genome zipper from corresponding chromosomes. Category refers to the organ categories based on AE-relatedness (blue) and un-relatedness (green).
